# The role of ChatGPT in clinical practice: perceptions, expectations and future implications amongst the clinical faculty

**DOI:** 10.12669/pjms.41.9.12131

**Published:** 2025-09

**Authors:** Laiba Zahir, Laloona Hussian, Wahab Zia, Iqbal Haider

**Affiliations:** 1 Laiba Zahir Medical Undergraduate, Khyber Medical College, Peshawar, Pakistan; 2 Laloona Hussian Medical Undergraduate, Khyber Medical College, Peshawar, Pakistan; 3 Wahab Zia House Physician, Khyber Teaching Hospital, MTI/KTH, Peshawar, Pakistan; 4 Iqbal Haider Professor of Medicine, Khyber Teaching Hospital, MTI/KTH, Peshawar, Pakistan. Khyber Teaching Hospital, MTI/KTH, Peshawar, Pakistan

**Keywords:** Artificial Intelligence, Clinical Decision Support System, Qualitative Research, Healthcare Technology

## Abstract

**Background & Objective:**

Artificial intelligence (AI) is transforming various industries, including healthcare. ChatGPT, introduced by OpenAI, is an advanced AI model with potential applications in medical practice. However, its reliability and effectiveness in clinical settings remain under investigation. This study explored the perspectives of healthcare professionals regarding the integration of ChatGPT in clinical practice, focusing on perceptions, expectations, and implications.

**Methodology:**

A qualitative study was conducted at Khyber Teaching Hospital, Peshawar from 15^th^ September 2023 till 30^th^ June 2024, using semi-structured interviews with a total of 17 clinical faculty members. The study primarily utilizes an inductive methodology, concentrating on the extraction and analysis of the contextual insights obtained from comprehensive, face-to-face interviews conducted with consultants in diverse medical fields at Khyber Teaching Hospital. Thematic analysis was performed to identify key insights.

**Results:**

Clinicians demonstrated varied awareness of ChatGPT, with some utilizing it for research purposes while others remained unfamiliar. Concerns regarding credibility, ethical considerations, and the potential for AI-driven misinformation were highlighted. While ChatGPT was viewed as a supportive tool in clinical workflows, reliance on AI for decision-making was questioned. Future integration requires structured implementation, training, and regulatory measures for AI. Participants emphasized the need for structured implementation, which includes safe incorporation of ChatGPT into clinical practice, formal training should enhance digital literacy and critical evaluation skills. AI tools should be incorporated into electronic medical records (EMRs) with real-time clinician oversight. A compact ethical and legal guidelines should be made to define responsibility, and pilot testing in controlled settings.

**Conclusion:**

ChatGPT holds promise for clinical practice, yet its adoption necessitates cautious implementation, clinician training, and regulatory oversight to ensure safe and ethical usage.

## INTRODUCTION

Artificial intelligence is becoming a revolutionary force across the globe. This innovative technology, in the form of a digital computer software or a machine, is capable of performing intelligent tasks that remarkably resemble those of a human being. Artificial intelligence systems are formulated to gather insights from their environment and make decisions based on the data they obtain.^1^ Chat bots, for example, are computer programs that are able to mimic human exchange through different modes of connectivity, including voice, text and visuals.^2^ The advancement of chatbots has led to major transformations across various aspects of our lives. In 2018 and 2019, the first two versions of the generative pretrained transformer (GPT), which are based on natural language processing (NLP) technology, were introduced. Our research is particularly focused on the artificial intelligence software ChatGPT.

Chat Generative Pre-Trained Transformer (ChatGPT) was introduced by OpenAI in 2019 with its foundation laid in 2015 by a team of researchers, including Elon Musk and Sam Altman. It garnered immediate attention due to its impressive text and image generation abilities. ChatGPT operates on a large-scale pre-trained language model enabling it to swiftly and precisely understand customer inquiries and produce responses that sound natural.^1^

ChatGPT is seen as a promising tool in future healthcare; its ability to store massive amounts of information could benefit practitioners in making diagnoses and decisions based on a patients’ data and history. ChatGPT can appropriately interpret patients’ feedback, extract relevant information, and propose provisional or preliminary diagnoses, medico-surgical advices and essential diagnostic test schemes by utilizing a pattern learning methodology based on extensive medical literature.^3^

ChatGPT can summarize a patient’s medical record and medical history, hence maintaining a well-stored medical bio-data record. Moreover, ChatGPT has the ability to provide reminders about medication intake, suggest appropriate dosage, details regarding possible side effects, interactions between drugs, and other vital elements of medication management.^4^

With the help of its natural language processing abilities, ChatGPT can be trained to evaluate and decipher medical images such as ultrasounds, MRIs and X-rays and acknowledge the structures and features within these images.^5^ This could drastically lessen the workload on medical professionals and radiologists by facilitating the swift and precise identification of diseases, injuries and anomalies.^5^ With functionalities that include self-operating extraction of electronic medical records, it is understood that technologies like ChatGPT possess the capability to revolutionize medicine.^6^ Although ChatGPT displays exceptional outputs, its ability in handling real-world problems and situations, especially in fields such as medicine which require intricate cognitive functions, remains uncertain.

The sole driving force behind conducting this research was to know the detailed knowledge of healthcare professionals’ regarding the role of ChatGPT in clinical practice. We also aimed to know about the generalized perception, the future expectations and the measures which our healthcare professionals’ will take in clinical practice regarding the beneficial role of ChatGPT. By knowing the viewpoints of medical practitioners regarding the application of ChatGPT in clinical practice might reveal important information about the effects of implementing this technology in healthcare sector. Acknowledging these factors, this study can shed light on possible advantages and limitations of ChatGPT in clinical practice.

## METHODS

This qualitative study was conducted from 15^th^ September 2023 till 30^th^ June 2024. Thematic analysis was performed following AMEE Guide No. 133 Guidelines.^7^

### Ethical approval:

Approval was obtained from the Institutional Review and Ethics Board (IREB) on First of September, 2023 (Ref.#523/DME/KMC; dated: September 1, 2023) according to the declarations of Helsinki.^8^

Using a qualitative research approach, this study explores medical professionals’ perspective of the utility of the ChatGPT in clinical practice. A focus group approach is well suited for investigating the viewpoints of consultants from different fields, which facilitates the deep understanding of the subject. The primary study methodology is inductive with the goal of extracting themes and patterns from the information gathered from the in-person, comprehensive interviews of consultants in different medical and surgical fields. This method helps to generate detailed perspectives on the perceived advantages, difficulties, and medicolegal issues related to incorporate ChatGPT into clinical practice. Participants with experience or skills in clinical practice and familiarity with the application of AI technology in healthcare were chosen through the use of a purposive sampling approach. By ensuring that the chosen participants can offer helpful feedback on the study question, this sampling approach improves the breadth and depth of the data gathered. Data were collected via in-person, comprehensive interviews with medical experts at Khyber Teaching Hospital Peshawar, Pakistan. The questionnaire for the interviews was validated through pilot testing and Focus group discussion of subject experts. The interviews were recorded and transcribed, and thematic analysis was used to examine the interview-derived qualitative data. All interviews were of an 8-12 minutes. The Correspondence author, being a professor of medicine at Khyber teaching hospital, Peshawar and medical educationist developed the themes and subthemes for analysis in liaison with subject expert focus group discussions. The inclusion criteria for the study were consultants in the fields of medicine, gynecology, surgery, and pediatrics. Participants were informed and consented before conduction of interviews. Interviews were conducted in the offices of the respective participants. All residents, house officers and undergraduates were excluded.

## RESULTS

### Awareness and familiarity:

While some clinicians were aware of ChatGPT through academic exposure, others had no prior knowledge.

### Perceived Usefulness:

Many clinicians acknowledged ChatGPT’s potential in administrative tasks, research, and preliminary decision-making but raised concerns about its reliability in direct patient care.

### Trust and Credibility:

The model’s dependency on outdated training data and lack of medical validation were major concerns.

### Obstacles and Concerns:

Barriers included lack of technical proficiency, resistance to AI-driven decision-making, and ethical challenges.

### Medico-Legal Implications:

Clinicians highlighted concerns regarding data security, patient privacy, and accountability in AI-assisted diagnoses.

**Fig.1:**
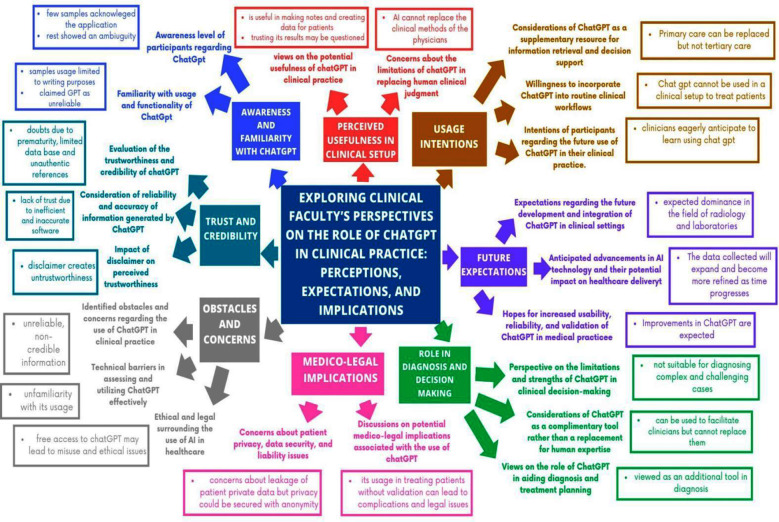
Conceptual framework.

Fig.1 elaborates a conceptual framework; Table-I elicits Clinician’s perspective on ChatGPT Thematic summary along with full quotes while Table-II is highlighting all themes along with subthemes.

**Table-I:** Clinician’s Perspective on ChatGPT Thematic Summary with Full Quotes

Theme	Summary	Quotes
1. Awareness & Familiarity	ChatGPT is relatively new to many clinicians. Only two had significant awareness and hands-on experience, mainly for academic tasks.	“Yes, I have heard about ChatGPT and currently it is on the news and on social media as well, and I have used it for some of our assignments and some of our tasks; it was truly helpful.” — (P-1) “I use it for medical writing purposes. I use it for writing introductions, but I have not used it for patient treatment plans or anything like that. Currently, as the ChatGPT itself says, they are not up-to-date, and they have provided information through 2021. So currently, I do not recommend using ChatGPT for treating patients.” — (P-5)
2. Perceived Usefulness in Clinical Setup	Mixed opinions. Some find it helpful for documentation and follow-up, but others fear its clinical application due to lack of human reasoning.	“In the clinical setup, yes, you can use it to write your notes, which can save your time… but again, I would say if you trust whatever has been generated by ChatGPT, you might be wrong in doing it. Therefore, what I do is I use it; it generates something for me, it saves my time, and then I review it again. Therefore, the point is that AI cannot replace your own intelligence.” — (P-6) “Yes, in the clinical setting, although we are not accustomed to its usage as such, I expect a lot… but I fear we may lose many lives because of use of this GPT because its thinking capacity is not like a human brain, so sometime it may make disaster in some decisions…” — (P-2)
3. Trust & Credibility	Many clinicians expressed concern over the reliability and accuracy of ChatGPT due to its lack of verified references and disclaimers.	“The trust and credibility depend upon the references from where they have generated the data or from where they have taken the data. Therefore, according to the reference, if it is from an authentic source, then its credibility is proven. However, if it is not from the credible source, then we cannot trust it.”—(P-1) “Well, there is always a disclaimer at the start of any software and ChatGPT, as I have primarily seen it; it gives a disclaimer that we are not responsible for any side effect or harm that can be done. Therefore, you cannot trust it.” — (P-11)
4. Obstacles & Concerns	ChatGPT’s polished language may mask inaccurate or irrelevant content. Generational tech gaps and vague sourcing are also key concerns.	“Concern is that they provide you a very good answer to anything, but you do not know that the answer is right or wrong… the content was not right… but the presentation was perfect, so I definitely doubt it.”—(P-4) “The obstacles would be the generation gaps that we have… they will be depending on the younger generation. That is the obstacle and issues.”— (P-10)
5. Medicolegal Implications	There are legal and ethical concerns around misdiagnosis, patient harm, and data privacy. Use without validation is viewed as dangerous.	“Medicolegal implications can be there if a person is just blindly following the ChatGPT as far as patient treatment is concerned… you will be facing a medicolegal issue…”— (P-5) “The medico-legal implications are patient privacy; patient data can be manipulated by many others… then there is possible use for it.”— (P-1)
6. Role in Diagnosis & Decision Making	Some believe ChatGPT can assist with differential diagnosis but warn it cannot replace human expertise, especially in emotional or complex scenarios.	“We can use it for differential diagnosis… if it helps in day-to-day cases… I would definitely use it…”—(P-9) “I think self-diagnosis is possible, but it will create many problems in the community… suffer from certain side effects…”—(P-4) “Initially, if you provide this tool with proper data, it can list your differential diagnosis… it will assist you, but I do not think it can replace a clinician or a physician.” — (P-8)
7. Future Expectations	There’s cautious optimism about ChatGPT’s role in diagnostics, data analysis, and radiology—as long as reliability and supervision improve.	“I think time will tell a lot of things, especially in radiology, it will take over… it is something that has come, we have to learn it…”—(P-7) “Maybe I think in the future, it may improve… The trust which is not there, might improve with time.” — (P-9)
8. Usage Intentions	Opinions are split—some are open to using it as a supportive tool, while others reject it due to fears of shortcuts, misuse, and oversimplification.	“No, legally it cannot be… ChatGPT is kind of shortcut so, a proper clinician will never go for shortcut.”—(P-10) “As I said, it can first replace the outskirt, the primary care… but replacing complicated diseases… it’s not a thing of the near future.” — (P-6)

**Table-II:** Themes and subthemes.

Themes	Sub themes
1. Awareness And familiarity with ChatGPT	Awareness level of participants regarding ChatGPT Familiarity with usage and functionality of ChatGPT
2. Perceived usefulness in clinical setup	Views on the potential usefulness of ChatGPT in clinical practices Concerns about the limitations of ChatGPT in replacing human clinical judgment
3. Trust and credibility	Evaluation of trustworthiness and credibility of ChatGPT Consideration of reliability and accuracy of information generated by ChatGPT Impact of disclaimer on perceived trustworthiness
4. Obstacles and concerns	Identified obstacles and concerns regarding the use of ChatGPT in clinical practice Technical barriers in accessing and utilizing ChatGPT effectively Ethical and legal surrounding use of AI in healthcare
5. Medico-legal implications	Discussions potential medico-legal implications associated with the use of ChatGPT Concerns about the patient privacy, data security, and liability issues
6. Role in diagnosis and decision making	Views on the role of ChatGPT in aiding diagnosis and treatment planning Perspective on the limitations and strengths of ChatGPT in clinical decision making Considerations of ChatGPT as a complimentary tool rather than a replacement for human expertise
7. Future expectations	Expectations regarding future development and integration of ChatGPT in clinical settings Anticipated advancement in AI technology and their potential impact on healthcare delivery Hopes for increased usability, reliability, and validation of ChatGPT in clinical practice
8. Usage intentions	Intention of participants regarding the future use of ChatGPT in their clinical practice Willingness to incorporate ChatGPT into routine clinical workflows Considerations of ChatGPT as a supplementary resource for information retrieval and decision support

## DISCUSSION

The study’s findings align with existing literature, emphasizing both the potential and limitations of ChatGPT in clinical practice. AI-driven healthcare models have been widely debated regarding their role in improving efficiency while ensuring ethical compliance. Previous studies indicate that ChatGPT can assist in drafting medical documentation and patient education materials, yet its lack of human judgment limits its ability to replace clinicians.

### Contribution to Medical Literature and Importance of the study:

Our survey revealed that although most of the clinicians were aware of ChatGPT, they confined its application majorly to research writing. These findings align with previous studies highlighting ChatGPT’s primary use in academic and scientific work rather than direct clinical applications.^9^ Another key concern identified in our study was trust and credibility. Where ChatGPT is nearly adequate in generating answers for simple case studies, when provided with complex cases such as surgical complications and nasal cancer prognosis, the results are deemed unsatisfactory and unreliable, which further calls into question the capability and credibility of ChatGPT.^10^ Despite these challenges, clinicians acknowledged AI’s potential in diagnosis and decision-making.^11^ ChatGPT can assist in a literature review, interpreting investigations, and generating differential diagnoses.^12^ The convenience of obtaining preliminary information before a visit to a physician not only reduces the mental stress experienced by patients but also facilitates effective counselling and informed decision-making by the physician.^13^

### Clinical relevance:

Although the final say of any medical decision should always be a health professional, ChatGPT may be used to help provide support and suggestions for treatment based on patient symptoms and medical history.^14^ Lastly, ChatGPT may not be able to provide empathy and emotional support to patients in the same way that a human healthcare provider can.^15^ Although the final say of any medical decision should always be a health professional, ChatGPT may be used to help provide support and suggestions for treatment based on patient symptoms and medical history. The future prospects of ChatGPT in medicine are extensive, spanning research, imaging, pharmacology, and patient management.^16^ From a medico-legal aspect, ChatGPT raises alarming concerns regarding data privacy, accountability and bias. The large data-set used for the algorithmic learning of ChatGPT is reflective of the inbuilt biases and increased risk of discriminatory medical guidance.^17^,^18^ In specialties, such as urology, patient privacy is a major concern as patient’s sensitive information is involved.^13^

To reduce the risk of such sensitive data being leaked or misused, the need for strong AI governance, ethical monitoring, and regulative policies to safeguard patient’s privacy and confidentiality is of utmost importance.^19^ AI-driven hospitals demonstrate that structured AI integration can enhance medical practice, optimizing workflow efficiency and reducing human error.^20^

Our findings align with the literature suggesting ChatGPT’s potential in clinical documentation, patient engagement, and trial analysis.^21^ ChatGPT can clearly help the healthcare industry by providing a more objective and evidence-based approach to decision-making, reducing the risk of human error on the basis of its unparalleled speed of information processing.^21^,^22^ However, the practical implementation of ChatGPT in resource-limited settings remains a challenge due to infrastructure constraints, lack of regulatory frameworks, and the digital divide. In conclusion, the future of AI in healthcare looks promising. Still, it is important to approach its adoption and implementation with caution and care in order to maximize the potential benefits it can bring to patient care.^22^

### Strength of the study:

In-depth face to face interviews were conducted to explore the view points of the clinicians regarding the implementation of ChatGPT into clinical setup.

### Limitations:

The major limitations of this research are:

1. Limited Sample Size: The study included a specific group of clinicians, minimizing generalizability. However, this is the underlying philosophy of qualitative research work.
2. Short-Term Analysis: Longitudinal impact assessments were not conducted.
3. Technological Barriers: Some participants lacked experience with AI, affecting responses.

## CONCLUSION

ChatGPT presents promising applications in healthcare, particularly in administrative and research domains. However, concerns regarding reliability, ethical considerations, and regulatory oversight must be addressed. Structured training programs and policy development are essential for the responsible integration of UpToDate Version of AI in clinical practice.

### Actionable Recommendations:

1. Training programs for clinicians unfamiliar with AI tools.
2. Ethical guidelines to be established to address concerns related to patient privacy and data security.
3. Regulatory policies to monitor AI applications in healthcare with Up-to-date version.

### Areas for future research:

Further research is required to analyze the long-term impact of ChatGPT in clinical decision-making.
